# Low serum magnesium concentrations are associated with a high prevalence of premature ventricular complexes in obese adults with type 2 diabetes

**DOI:** 10.1186/1475-2840-11-23

**Published:** 2012-03-09

**Authors:** Liana C Del Gobbo, Yiqing Song, Paul Poirier, Eric Dewailly, Ronald J Elin, Grace M Egeland

**Affiliations:** 1 School of Dietetics & Human Nutrition, McGill University, 21,111 Lakeshore Road, Ste. Anne de Bellevue, Quebec, H9X 3V9, Canada; 2 Division of Preventive Medicine, Brigham and Women's Hospital, 900 Commonwealth Ave., Boston, Massachusetts, 02215, USA; 3 Quebec Heart and Lung Institute, Sainte-Foy, Quebec, Canada; 4 Public Health Research Unit, CHUL Research Center, Centre Hospitalier, Universitaire de Quebec Sainte-Foy, Quebec City, Quebec, G1V 5B3, Canada; 5 Department of Pathology and Laboratory Medicine, University of Louisville, Louisville, Kentucky, USA 40292

**Keywords:** Magnesium, Hypomagnesemia, Premature ventricular complexes, Premature ventricular beats, Ectopic beats, Arrhythmia, Type 2 diabetes

## Abstract

**Background:**

Premature ventricular complexes (PVC) predict cardiovascular mortality among several adult populations. Increased arrhythmia prevalence has been reported during controlled magnesium (Mg) depletion studies in adults. We thus hypothesized that serum magnesium (sMg) concentrations are inversely associated with the prevalence of PVC in adults at high cardiovascular risk.

**Methods:**

Anthropometric, demographic and lifestyle characteristics were assessed in 750 Cree adults, aged > 18 yrs, who participated in an age-stratified, cross-sectional health survey in Quebec, Canada. Holter electrocardiograms recorded heart rate variability and cardiac arrhythmias for two consecutive hours. Multivariate logistic regression was used to evaluate the associations between sMg and PVC.

**Results:**

PVC prevalence in adults with hypomagnesemia (sMg ≤ 0.70 mmol/L) was more than twice that of adults without hypomagnesemia (50% vs. 21%, *p *= 0.015); results were similar when adults with cardiovascular disease history were excluded. All hypomagnesemic adults with PVC had type 2 diabetes (T2DM). Prevalence of PVC declined across the sMg concentration gradient in adults with T2DM only (*p *< 0.001 for linear trend). In multivariate logistic regressions adjusted for age, sex, community, body mass index, smoking, physical activity, alcohol consumption, kidney disease, antihypertensive and cholesterol lowering drug use, and blood docosahexaenoic acid concentrations, the odds ratio of PVC among T2DM subjects with sMg > 0.70 mmol/L was 0.24 (95% CI: 0.06-0.98) *p *= 0.046 compared to those with sMg ≤ 0.70 mmol/L.

**Conclusions:**

sMg concentrations were inversely associated with the prevalence of PVC in patients with T2DM in a dose response manner, indicating that suboptimal sMg may be a contributor to arrhythmias among patients with T2DM.

## Background

Premature ventricular complexes (PVC) are a relatively common electrocardiographic abnormality presenting in individuals with and without overt cardiovascular disease. In the latter case, PVC pathogenesis has traditionally been considered idiopathic and in the absence of severe clinical symptoms or structural cardiac abnormalities, their presence benign [1,2]. Recent prospective studies evaluating the prognostic significance of PVC for sudden and total cardiac death in apparently healthy adults directly challenge this view. Among individuals without history of heart disease or stroke, PVC counts independently predicted future cardiac events or sudden cardiac death compared to those without PVC [3-7]. These findings are consistent with hypothesis that PVC may indicate electrical instability and increased susceptibility to ventricular fibrillation [6], the most common cause of sudden cardiac death.

Mg^2+ ^antagonizes calcium on the atrioventricular node [8] and myocardial Mg^2+ ^deficiency decreases intracellular potassium, resulting in a less negative resting membrane potential and enhanced vulnerability to ventricular arrhythmia [9-11]. Two randomized, double-blind crossover studies by USDA scientists showed that reducing intake of dietary magnesium (Mg) in postmenopausal women in a metabolic unit to 33-50% of the Mg recommended dietary allowance can induce heart rhythm changes [12,13]. During the Mg depletion phase, calcium, potassium, copper and other nutrients were concomitantly supplemented [12] and arrhythmias were relieved by Mg supplementation, suggesting that Mg insufficiency resulting from a diet that would not be considered an atypical Western menu can induce arrhythmias in older women.

In adults from the Framingham Offspring cohort free of clinically apparent heart disease, total serum magnesium (sMg) was significantly inversely associated with prevalence of complex or frequent PVC after adjustment for multiple covariates, including serum potassium [14]. As type 2 diabetes (T2DM) is the most common condition associated with low sMg or hypomagnesemia (sMg ≤ 0.70 mmol/L) [15] and T2DM significantly increases risk of PVC and sudden cardiac death [16], the association between sMg and PVC may be modified by diabetic status. However, testing for such potential effect modification has not been reported previously.

We hypothesized that sMg concentrations are inversely associated with the prevalence of PVC in a general adult population at high cardiovascular risk, James Bay Cree, and tested for effect modification by T2DM. Mg intakes in Cree adults, particularly Cree men, are inadequate, with 93% of men 19-30 yrs and 100% of men ≥ 31 yrs consuming less than the Mg recommended dietary intake in some communities [17]. Due to rapid dietary and lifestyle transition and consequent increasing obesity rates, prevalence of T2DM among Cree is among the highest in the world [18].

## Methods

A subset of adults (≥ 18 yrs) of Cree descent from the James Bay region of Quebec, Canada were selected from a comprehensive aboriginal health survey 'Nituuchischaayihitaau Aschii: A Multi-Community Environment and Health Longitudinal Study in Iiyiyiu Aschii.' This cross-sectional study included fasting participants from seven communities sampled between 2005-2009 for which sMg, covariates and 2-hr Holter electrocardiogram recordings were available. Bilingual Cree were trained to conduct interviews and recruit participants. Ethics for the health survey was obtained from the Cree Board of Health and Social Services of James Bay (CBHSSJB), Centre Hospitalier Universitaire de Québec (Laval), and McGill universities. A research agreement with communities was developed with the Cree Board of Health and all individual study participants provided informed written consent in Cree or English.

A total of 834 adults were assessed for anthropometric measures, demographic and lifestyle variables (smoking, alcohol consumption, exercise); sMg, full medical history, and Holter data (PVC) were available for 750 adults included in analysis. Individuals with missing values did not differ significantly in covariate distribution from included individuals.

### Variable assessment

Anthropometric variables were measured without shoes by trained nurses. Height, measured in centimeters using a graduated tape, and weight, determined using a foot-to-foot bioelectrical impedance instrument (Tanita Corp, Arlington Heights, IL, USA), were combined to obtain body mass index (BMI). Exercise (frequency in which individuals reported to engage in vigorous physical activity for at least 20 min at a time) was assessed by a culturally adapted, short version of the International Physical Activity Questionnaire validated in Cree adults [19]. Smoking status (current, former or never) and alcohol consumption (≥ 3 drinks/day, < 3 drinks day, or never, of standard drinks of beer, liquor, wine, mixed drinks, or shooters) were self-reported. As cellular and animal models, epidemiological studies and clinical trials suggest that long-chain polyunsaturated fatty acids exert anti-arrhythmic effects [20,21], we tested the potentially modifying effects of plasma eicosapentaenoic acid (EPA) and docosahexaenoic acid (DHA) as nutritional covariates in models. Medical charts for each participant were reviewed and detailed cardiovascular event history or conditions, metabolic conditions, cardiovascular drugs/supplement use, and selected other conditions were recorded. In characterizing the metabolic syndrome (MetS), the consensus definition was used [22]. Adults with T2DM had previously been diagnosed or presented with fasting glucose (FG) concentrations ≥ 7 mmol/L.

Serum Mg was determined at Trace Elements Laboratory the London Laboratory Services group, London, Ontario, Canada using the colorimetric endpoint method with addition of xylidyl blue. Mg content was measured photometrically as a function of the decrease in xylidyl blue absorbance (laboratory reference range: 0.65-1.05 mmol/L). For the determination of EPA and DHA concentrations, 200-μL aliquots of plasma were extracted after the addition of chloroform: methanol (2:1), in the presence of a known amount of internal standard (diheptadecanoyl phospholipid). Total phospholipids were isolated from the lipid extract via thin-layer chromatography with the use of developing solvent heptane:isopropyl ether:acetic acid (60:40:3). Transmethylation with boron trifluoride and methanol preceded capillary gas-liquid chromatography to determine fatty acid concentrations.

Frequency of premature ventricular beats, distinct from supraventricular beats, were derived from a 2-hour Holter monitoring system (GE Marquette Series 8500) with a recording frequency of 128 Hz. Seven leads (derivations V5, V1, and AVF) were installed when subjects arrived to the clinic. During the 2-hour recording, subjects remained at the clinic, completing anthropometric measurements and lifestyle questionnaires.

### Statistical approach

PVC prevalence for all adults ≥ 18 yrs (n = 750) on 2-hour Holters was presented stratified by demographic, anthropometric and lifestyle variables. We considered ≥ 1 PVC/hr and > 6 PVC/hr recording as endpoints in this high cardiometabolic risk sample; elevated risk for sudden cardiac death was reported for high-risk participants in the Cardiovascular Health Study with > 153 PVC over 24 h, or about 6 events/hr [23].

Unadjusted prevalence of PVC across sMg groupings for the entire sample and subgroups (with cardiovascular event history or conditions excluded, those with T2DM, and MetS) was determined. Frequencies were compared using *X *^2 ^tests or Fisher's exact test when sample sizes in contingency tables were small. Odds ratios and 95% confidence intervals using multivariate logistic regression for ≥ 1 PVC/hr and > 6 PVC/hr across the sMg concentration range for the whole population were determined for five different covariate models. Hypomagnesemia was defined as sMg ≤ 0.70 mmol/L. In subgroup analysis for adults with type 2 diabetes and no prior cardiovascular disease (CVD) (n = 149), only the odds ratio of ≥ 1 PVC/hr for normomagnesemic diabetics (sMg > 0.70 mmol/L) relative to the reference group of hypomagnesemic diabetics (sMg ≤ 0.70 mmol/L) were determined due to sample size constraints. For multivariate models, individuals with prior CVD were defined as those with one or more condition listed under "cardiovascular event history or conditions" or taking CVD drugs in Table 1; hypertensives were excluded from this definition. Additional sensitivity analysis was performed excluding adults with kidney disease, as this condition is associated with altered Mg homeostasis and sMg concentrations [24].

**Table 1:** Medical chart review of adults (≥ 18 yrs) of the general Cree population (n = 750)

*Cardiovascular event history or conditions*	*N*
Ischemia/infarction	2
Cardiac insufficiency	4
Atrial fibrillation	3
Ventricular arrhythmia/tachycardia	5
Bradycardia	3
Ventricular hypertrophy	2
Angina	6
Hypertension (diagnosed)	185
(BP > 130/85 mmHg, or treatment of diagnosed)	249
Ischemic heart disease	6
Diseases of the pulmonary circulation	3
Stroke	4
Cerebrovascular diseases	11
Diseases of arteries, arterioles or capillaries	6
*Cardiometabolic conditions*	
Metabolic syndrome	352
(excluding diagnosed type 2 diabetics/FG ≥ 7 mmol/L)	140
Gestational diabetes (history of)	23
Type 1 diabetes	1
Type 2 diabetes (diagnosed)	140
(diagnosed or FG ≥ 7 mmol/L)	176

*Cardiovascular drug/supplement use*	
Antiplatelet	6
Antihypertensives	183
Diuretics (specifically)	62
Cholesterol-lowering drugs	79
Statins (specifically)	73
Multivitamins	41
Mg supplements	8

*Hormonal or menstrual status*	
Post-menopausal	104
Hormone replacement therapy	1
Oral contraceptives	92
Currently breastfeeding	35

*Other*	
Kidney disease	13
Cancer	6

## Results

Our population included 441 women (58.8%) and 309 men (41.2%) with a mean age of 38.9 ± 14.8 yrs. Mean BMI was 33.9 ± 7.0 kg/m^2^. More than half of adults (51.3%) identified as current smokers. Hypertension was the most common CVD condition (33.2%) and antihypertensive drugs the most common class of CVD medications taken (24.4%). The percentage of adults with MetS was 46.9%; prevalence of T2DM (diagnosed or FG ≥ 7 mmol/L) was 23.5% (Table 1).

Prevalence and frequency of PVC differed by smoking status and was significantly higher in men, older participants, those with MetS or T2DM (*p *< 0.001 for each covariate). PVC prevalence varied significantly with alcohol consumption, exercise and CVD history (excluding hypertensives) (*p *< 0.05). There was a trend towards an association between PVC and hypertension (*p *< 0.10) but obesity was not significantly associated with PVC (*p *= 0.39) (Table 2) nor was diuretics use (*p *= 0.63).

**Table 2:** Characteristics of Cree adults (≥ 18 yrs) according to PVC prevalence during Holter electrocardiogram (n = 750)

Characteristic	No PVC n = 591	1-6 PVC/hr n = 118	> 6 PVC/hr n = 41
Sex			
*Male*	217 (37%)	64 (54%)	28 (68%)
*Female*	374 (63%)	54 (46%)	13 (32%)***
Age (yrs)			
*18-30*	194 (33%)	23 (19%)	5 (12%)
*30-50*	302 (51%)	44 (37%)	14 (34%)
*≥ 50*	95 (16%)	51 (43%)	22 (54%)***
BMI (kg/m^2^)			
*< 30*	170 (29%)	36 (31%)	8 (20%)
*≥ 30*	421 (71%)	82 (69%)	33 (80%)
Smoking			
*Current*	329 (56%)	40 (34%)	16 (39%)
*Former*	204 (34%)	61 (52%)	21 (51%)
*Never*	58 (10%)	17 (14%)	4 (10%)***
Alcohol^1^			
*< 3 drinks/d*	271 (46%)	48 (41%)	10 (24%)
*Never*	312 (54%)	69 (59%)	31 (76%)**
Exercise			
*≥ 4 times/wk*	187 (32%)	37 (31%)	13 (32%)
*1-3 times/wk*	217 (36%)	32 (27%)	10 (24%)
*None*	187 (32%)	49 (42%)	18 (44%)**
Hypertension	153 (26%)	41 (35%)	15 (37%)*
CVD history^1^	18 (3%)	7 (6%)	4 (10%)**
MetS^2^	95 (16%)	30 (25%)	15 (37%)***
Diabetes^3^	122 (21%)	37 (31%)	17 (41%)***

*p < 0.01 **p < 0.05 ***p < 0.001

^1 ^Includes history of cardiovascular events/arrhythmia or CVD, excludes hypertensives

^2 ^Excludes diagnosed type 2 diabetics/FG ≥ 7 mmol/L

^3 ^Includes diagnosed type 2 diabetes or FG ≥ 7 mmol/L

**Figure 1:**
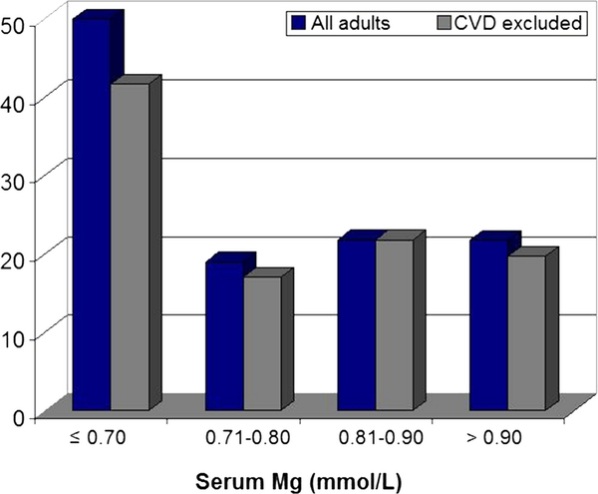
**PVC prevalence (%) during 2-hr Holters among adults (n = 750) according to sMg concentrations (mmol/L)^1^**. Legend: CVD excluded (grey bars) omits individuals with cardiovascular event history or conditions. Difference in PVC prevalence among participants with sMg ≤ 0.70 vs. > 0.70 mmol/L among all adults (blue bars): *p *= 0.015; for CVD excluded (grey bars): *p *= 0.07.

Unadjusted prevalence of PVC in adults with hypomagnesemia (sMg ≤ 0.70 mmol/L) was over twice that of adults without hypomagnesemia (50% vs. 21%, *p *= 0.015); results were not different when adults with cardiovascular disease history were excluded (Figure 1). In multivariate logistic regression analyses for all adults (n = 750), odds of ≥ 1 PVC/hr declined precipitously from the hypomagnesemic reference group to 0.24 (95% CI: 0.07-0.87) in those with sMg 0.71-0.80 mmol/L in the fully adjusted model (Model 5); risk did not substantively change at higher sMg concentrations (Table 3). Due to low sample size among the hypomagnesemic reference group, it was not possible to further define the sMg threshold associated with reduced odds ≥ 1 PVC in this sample. Model covariates had little influence on adjusted odds ratios; inclusion of EPA in any model had no impact on estimates and was excluded.

**Table 3:** Adjusted odds ratios & 95% confidence intervals for presence of ≥ 1 PVC on 2-hr Holters across the sMg concentration range (n = 750)

sMg (mmol/L)	≤ 0.7	0.71-0.80	0.81-0.90	> 0.90
Model 1	1.00	0.26 (0.07-0.88)	0.27 (0.08-0.93)	0.22 (0.06-0.81)

Model 2	1.00	0.25 (0.07-0.85)	0.26 (0.08-0.88)	0.19 (0.05-0.71)

Model 3	1.00	0.25 (0.07-0.85)	0.26 (0.08-0.88)	0.20 (0.05-0.73)

Model 4	1.00	0.24 (0.07-0.86)	0.25 (0.07-0.92)	0.19 (0.05-0.77)

Model 5	1.00	0.24 (0.07-0.87)	0.26 (0.07-0.93)	0.18 (0.04-0.73)

Model 1: adjusted for age and sex

Model 2: additionally adjusted for region and BMI

Model 3: additionally adjusted for smoking, physical activity, alcohol consumption

Model 4: additionally adjusted for history of cardiovascular events/disease, type 2 diabetes, kidney disease, antihypertensive and cholesterol lowering drug use

Model 5: additionally adjusted for blood DHA

Significantly decreased adjusted odds of > 6 PVC/hr were observed in those with sMg above a sMg 0.75 mmol/L threshold in the whole population for all covariate models (Additional file 1). Odds of > 6 PVC/hr for those with sMg 0.75-0.80 relative to those with < 0.75 mmol/L were 0.10 (95% CI: 0.02-0.46) in the fully adjusted model (Model 5). A sMg threshold of 0.75 mmol/L was selected for this endpoint because ORs were maximally reduced using this cutoff value (Additional file 2).

**Figure 2:**
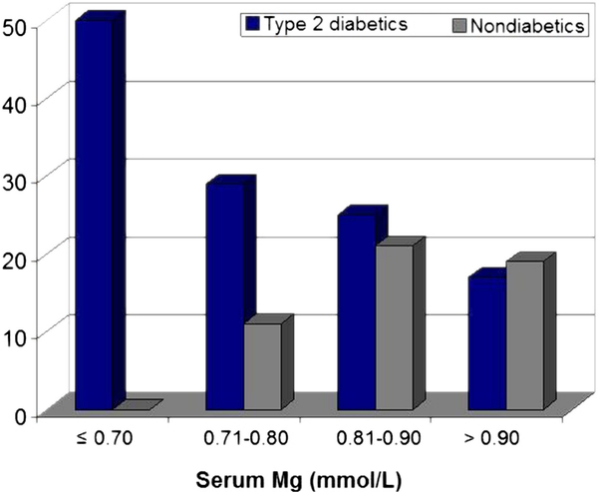
**PVC prevalence (%) during 2-hr Holters among adults without CVD (n = 652) by sMg concentrations (mmol/L) and diabetic status^1^**. Legend: PVC prevalence declined with sMg concentration in adults with T2DM (blue bars) only (*p *< 0.001 for trend), even when hypomagnesemic adults were excluded (*p *= 0.005 for trend). While no nondiabetic (grey bars) had PVC during monitoring, only 1% of nondiabetics were hypomagnesemic (sMg ≤ 0.70 mmol/L)

Further evaluation of the characteristics of hypomagnesemic subjects in this population revealed that all individuals with PVC had T2DM. When participants were stratified by diabetic status, prevalence of PVC declined across the sMg concentration gradient in adults with T2DM only (*p *< 0.001) (Figure 2). Decline in PVC prevalence with increasing sMg among participants with T2DM was significant even when hypomagnesemic adults were excluded (*p *= 0.005). Addition of diabetic medication use (metformin, gluconorm, or insulin) as covariates had no influence on model estimates. While only 1% of subjects without T2DM were hypomagnesemic (n = 5), none of these subjects had PVC during recording. In the subgroup with MetS but without T2DM, no significant difference in PVC prevalence across the sMg concentration gradient was observed (*p *= 0.84).

Among adults with T2DM and no CVD history (n = 149), individuals without hypomagnesemia (sMg > 0.70 mmol/L) had significantly lower risk of PVC on 2-hr Holter recording in fully adjusted models [OR 0.24 (95% CI: 0.06-0.98)] (Table 4). Sensitivity analysis excluding remaining individuals with kidney disease (n = 4 without CVD history) had no influence on model estimates.

**Table 4:** Effect of hypomagnesaemia in type 2 diabetics without CVD history on adjusted odds ratios & 95% confidence intervals for ≥ 1 PVC on 2-hr Holters (n = 149)

	Type 2 diabetics sMg ≤ 0.70 mmol/L	Type 2 diabetics sMg > 0.70 mmol/L
Model 1	1.00	0.28 (0.08-0.98)

Model 2	1.00	0.25 (0.07-0.92)

Model 3	1.00	0.26 (0.07-1.04)

Model 4	1.00	0.25 (0.06-1.03)

Model 5	1.00	0.24 (0.06-0.98)

Model 1: adjusted for age and sex

Model 2: additionally adjusted for region and BMI

Model 3: additionally adjusted for smoking, physical activity, alcohol consumption

Model 4: additionally adjusted for kidney disease, antihypertensive and cholesterol lowering drug use

Model 5: additionally adjusted for blood DHA

## Discussion

### Hypomagnesemia & ventricular arrhythmias

Our finding of significantly elevated PVC prevalence in adults with hypomagnesemia relative to adults without reduced sMg is consistent with early clinical studies reporting ventricular fibrillation and tachycardia in hypomagnesemia [25,26]. Hypomagnesemia is an established risk factor for polymorphic ventricular tachycardia torsades de pointes [27] and intravenous magnesium sulfate remains first-line therapy for tosades de pointes associated with long QT interval according to current ACC/AHA/ESC guidelines [28]. It has been suggested that the trigger for torsades de pointes are PVC resulting from an early afterdepolarization generated during the abnormally prolonged repolarization phase of affected myocardium [29].

Reports of increased prevalence of ventricular arrhythmias in individuals with low sMg concentrations have been criticized as lacking in evidence that the effect is attributable to Mg alone as opposed to a combination of hypomagnesemia and hypokalemia [30]. As we did not measure serum potassium in our cross-sectional design, this study provides no evidence to rebuke this claim, particularly as hypomagnesemia and hypokalemia frequently coexist and have similar etologies. In the Framingham Offspring cohort, apparently the only other general population study associating sMg with PVC, multivariate logistic regression analyses revealed that both serum potassium and Mg concentrations, when simultaneously entered into models, were inversely associated with complex or frequent PVC occurrence (*p *< 0.04). Each standard deviation decrement in potassium (0.48 mmol/L) or Mg (0.08 mmol/L) was associated with a similar increase in odds PVC [27% (95% CI 6-51%) for potassium, and 20% (95% CI 3-41%) for Mg] [14]. From physiologic studies, Mg is an established regulator of major ion channels in the cardiovascular system, including potassium, calcium, sodium, and others [31]. Potassium is not effective in the suppression of cardiac arrhythmia occurring in the presence of normal serum potassium; by contrast, Mg can attenuate arrhythmias regardless of sMg concentration [30,32,33]. Irrespective of the degree to which prevalence of PVC may differ due to sMg concentration independent of other electrolytes, this study supports hypomagnesemia as a risk factor for ventricular arrhythmias.

### Magnesium & arrhythmias: T2DM as an effect modifier

Low serum Mg has been associated with T2DM [34], but not metabolic states preceding T2DM [35,36], a finding consistent with our data. In this sample, diabetic status, but not MetS, was a major determinant of very low sMg levels; 76% of all hypomagnesemic adults had T2DM. While increased fractional excretion of Mg at elevated insulin concentrations [37] may be at play in both MetS and T2DM, mechanisms for Mg loss specific to diabetes, such as renal Mg wasting secondary to osmotic diuresis during glucosuria [38], might be associated with comparatively greater reductions in sMg.

As T2DM is the most common condition associated with low sMg [15] and diabetes significantly increases risk of ventricular arrhythmias [16], there is theoretical basis for the hypothesis that T2DM might modify the association between sMg and PVC. Here, we provide the first supporting evidence that T2DM is an important effect modifier of the association between sMg and PVC, with reductions in PVC prevalence across the sMg gradient in T2DM only, driving the significant associations observed in the entire population. While the PVC outcome variable (> 30 PVC/hr, multiform or repetitive) in the Framingham Offspring cohort was different than endpoints assessed here and T2DM prevalence was high in our population, whether the significant association between sMg and PVC risk reported in the Framingham cohort [14] might be modified by inclusion of T2DM status as a covariate is unknown. Replication studies and explicit testing for potential effect modification by diabetes in future analyses is required.

In this work, risk of > 6 PVC/hr was maximally reduced above a sMg threshold (0.75 mmol/L) within the normal sMg concentration range and prevalence of PVC declined across the normal sMg concentration gradient in adults with T2DM. In other populations, significantly reduced risk of ventricular arrhythmia [14], sudden cardiac death [39] and cardiovascular disease death [40,41] at elevated sMg concentrations have been reported. Thus, the findings of this study are consistent with evidence from other cross-sectional and prospective cohort studies reporting cardiovascular risk stratification within the normal sMg range, with reduced risk of both intermediary adverse outcomes and hard endpoints at higher sMg concentrations.

### Limitations

Limitations of this study include use of sMg instead of ionized Mg as a biomarker [26]; free intracellular Mg is the fraction regulating enzyme pathways [27]. Although sMg may not be a sensitive marker in reflecting mild Mg inadequacy or the intracellular Mg pool, further investigations evaluating the utility of sMg, an inexpensive and simple measure, as a cardiovascular risk biomarker are appropriate.

Consistent cut-offs for the number or complexity (multiforms, pairs, runs, R-on-T) of PVC per unit time of Holter recording associated with mortality risk across population groups of different ages, ethnicities or underlying cardiovascular risk have yet to be established [3-6]. Thus, a main limitation of this work is the uncertain prognostic significance of PVC endpoints selected in this high-risk population, which can be determined through long term follow-up for incidence of ventricular fibrillation, tachycardia, sudden cardiac death, or CVD mortality. Other limitations include the use of short-term (2 hr) Holter monitoring on a single occasion for PVC detection, and unknown left ventricular ejection fraction, the latter of which could have modulated prevalence of asymptomatic heart disease and PVC among patients with diabetes. Generalizability of our findings may be limited by the ethnic homogeneity of the sample and high prevalence of T2DM relative to most other general populations. Inclusion of other potentially relevant unmeasured covariates, such as serum potassium and other electrolytes, caffeine use, non-cardiovascular drugs, or stress/anxiety might reduce residual confounding. Consistency in sample demographics and CVD risk, monitoring environments, duration and specific PVC endpoints assessed in different populations will be important in reducing heterogeneity among future studies and facilitating comparisons.

## Conclusions

This study provides evidence that T2DM is an important effect modifier of the association between sMg and ventricular ectopy, with significant reductions in PVC prevalence across the sMg gradient in adults with T2DM. Diabetic status, but not MetS, was a major determinant of very low sMg levels in this population. PVC risk was particularly elevated in diabetic adults with hypomagnesemia, suggesting that future interventions to increase sMg concentrations among adults with T2DM may confer protection against ventricular arrhythmias. As hypomagnesemia is an under-diagnosed electrolyte abnormality common in T2DM [42], appropriately powered randomized controlled clinical trials are required to evaluate the potential of Mg to reduce arrhythmias and cardiovascular risk.

## Abbreviations

ACC: American College of cardiology; AHA: American heart association; ARIC: Atherosclerosis risk in communities; BMI: Body mass index; CVD: Cardiovascular event or conditions; CI: Confidence interval; CBHSSJB: Cree board of health and social services of James bay; DHA: Docosahexaenoic acid; EPA: Eicosapentaenoic acid; ESC: European society of cardiology; FG: Fasting glucose; HR: Hazard ratio; IDF: International diabetes federation; Mg: Magnesium; MetS: Metabolic syndrome; OR: Odds ratio; PVC: Premature ventricular complexes; sMg: Total serum magnesium; T2DM: Type 2 diabetes mellitus; USDA: United states department of agriculture.

## Competing interests

The authors declare that they have no competing interests.

## Authors' contributions

ED and GE conceived and designed the Cree health study and contributed to critical review of analysis and interpretation of data; YS, PP and RE critically reviewed the statistical design, analysis and interpretation of data; LD conducted the statistical analysis, drafted the manuscript and was responsible for its content. All authors read and approved the final manuscript.

## Supplementary Material

Additional file 1 **Adjusted odds ratios and 95% confidence intervals for presence of > 6 PVC/hr on Holter monitors across the sMg concentration range (n = 750)**. Click here for file

Additional file 2 **Odds ratios for the fully adjusted model (Model 5) for presence of > 6 PVC/hr on Holters are minimized using < 0.75 mmol/L sMg as the reference (n = 750)**. Click here for file
