# Supplementary material for: Low serum magnesium concentrations are associated with a high prevalence of premature ventricular complexes in obese adults with type 2 diabetes
Source: Cardiovasc Diabetol. 2012 Mar 9;11:23. doi: 10.1186/1475-2840-11-23 (PMC3337820; doi:10.1186/1475-2840-11-23)
Supplement: Additional file 1 — Adjusted odds ratios and 95% confidence intervals for presence of > 6 PVC/hr on Holter monitors across the sMg concentration range (n = 750). [file 1475-2840-11-23-S1.DOC]

**Additional file 1.** Adjusted odds ratios and 95% confidence intervals for presence of >6 PVC/ hr on Holter monitors across the sMg concentration range (n=750)

| sMg  (mmol/L) | **<0.75** | **0.75-0.80** | **0.80-0.85** | **0.85-0.90** | **>0.90** |
| --- | --- | --- | --- | --- | --- |
| Model 1 | 1.00 | 0.16 (0.05-0.54) | 0.41 (0.17-0.99) | 0.19 (0.07-0.53) | 0.15 (0.04-0.53) |
| Model 2 | 1.00 | 0.15 (0.04-0.53) | 0.39 (0.16-0.98) | 0.17 (0.06-0.49) | 0.15 (0.04-0.56) |
| Model 3 | 1.00 | 0.13 (0.04-0.45) | 0.36 (0.14-0.92) | 0.16 (0.05-0.46) | 0.13 (0.03-0.54) |
| Model 4 | 1.00 | 0.10 (0.02-0.45) | 0.37 (0.13-1.10) | 0.17 (0.05-0.57) | 0.10 (0.02-0.53) |
| Model 5 | 1.00 | 0.10 (0.02-0.46) | 0.38 (0.13-1.13) | 0.16 (0.05-0.57) | 0.06 (0.01-0.39) |

Model 1: adjusted for age and sex

Model 2: additionally adjusted for region and BMI

Model 3: additionally adjusted for smoking, physical activity, alcohol consumption

Model 4: additionally adjusted for history of cardiovascular events/disease, type 2 diabetes, kidney disease, antihypertensive and cholesterol lowering drug use

Model 5: additionally adjusted for blood DHA
