# Supplementary material for: Low serum magnesium concentrations are associated with a high prevalence of premature ventricular complexes in obese adults with type 2 diabetes
Source: Cardiovasc Diabetol. 2012 Mar 9;11:23. doi: 10.1186/1475-2840-11-23 (PMC3337820; doi:10.1186/1475-2840-11-23)
Supplement: Additional file 2 — Odds ratios for the fully adjusted model (Model 5) for presence of > 6 PVC/hr on Holters are minimized using < 0.75 mmol/L sMg as the reference (n = 750). [file 1475-2840-11-23-S2.DOC]

**Additional file 2**. Odds ratios for the fully adjusted model (Model 5) for presence of >6 PVC/ hr on Holters are minimized using <0.75mmol/L sMg as the reference (n=750)

| sMg reference group cutoff (x); OR 1.00 | **(x+0.1) - 0.80** | **0.80-0.85** | **0.85-0.90** | **> 0.90** |
| --- | --- | --- | --- | --- |
| < 0.76 | 0.20 (0.05-0.86) | 0.55 (0.19-1.61) | 0.24 (0.07-0.81) | 0.09 (0.01-0.57) |
| **< 0.75** | **0.10 (0.02-0.46)** | **0.38 (0.13-1.13)** | **0.16 (0.05-0.57)** | **0.06 (0.01-0.39)** |
| < 0.74 | 0.21 (0.06-0.79) | 0.46 (0.15-1.46) | 0.20 (0.06-0.73) | 0.07 (0.01-0.50) |
| < 0.73 | 0.30 (0.08-1.09) | 0.52 (0.15-1.75) | 0.23 (0.06-0.87) | 0.08 (0.01-0.58) |
